# DIMETER: A Haptic Master Device for Tremor Diagnosis in Neurodegenerative Diseases

**DOI:** 10.3390/s140304536

**Published:** 2014-03-07

**Authors:** Roberto González, Antonio Barrientos, Jaime del Cerro, Benito Coca

**Affiliations:** 1 Centre for Automation and Robotics UPM-CSIC, C/José Gutierrez Abascal 2, E-28006 Madrid, Spain; E-Mails: antonio.barrientos@upm.es (A.B.); j.cerro@upm.es (J.C.); 2 ETSIDI-UPM, C/Ronda de Valencia 3, E-28012 Madrid, Spain; E-Mail: benitomaria.coca.hernandez@alumnos.upm.es

**Keywords:** DIMETER, diagnostic tremor aids, Parkinson's disease (PD) diagnosis, essential tremor (ET) diagnosis, neurodegenerative diseases, haptic master, tremor device

## Abstract

In this study, a device based on patient motion capture is developed for the reliable and non-invasive diagnosis of neurodegenerative diseases. The primary objective of this study is the classification of differential diagnosis between Parkinson's disease (PD) and essential tremor (ET). The DIMETER system has been used in the diagnoses of a significant number of patients at two medical centers in Spain. Research studies on classification have primarily focused on the use of well-known and reliable diagnosis criteria developed by qualified personnel. Here, we first present a literature review of the methods used to detect and evaluate tremor; then, we describe the DIMETER device in terms of the software and hardware used and the battery of tests developed to obtain the best diagnoses. All of the tests are classified and described in terms of the characteristics of the data obtained. A list of parameters obtained from the tests is provided, and the results obtained using multilayer perceptron (MLP) neural networks are presented and analyzed.

## 1. Introduction

Many diseases have become growing problems in aging societies. Several neurodegenerative diseases, such as Parkinson's disease (PD), Alzheimer's disease, and sclerosis, can affect patients for long periods of time. Furthermore, the increase in life expectancy has resulted in the emergence of other natural (non-degenerative) disorders, such as essential tremor (ET), which cause severe movement disorders. Numerous studies have been conducted to measure or estimate the incidence and prevalence rates of both PD and ET (Table 1).

PD and ET are difficult to diagnose because there are many known movement disorders that can be confused with each other. The clinical manifestation of a disease may change because of previous treatment, the age of the subject (*i.e.*, possible dementia), the stage of the disease, the time of day, or drinking habits (*i.e.*, alcohol consumption). The correct diagnosis of patients with PD or ET can improve initial treatment and enable the tracking of a disorder in its initial stages.

Although neurodegenerative diseases can affect a great number of people for long periods of time, the increase in life expectancy can also lead to other degenerative disorders that are not as disabling as ET.

Different tests have been developed to evaluate the clinical manifestations of tremor. Unfortunately, although some of these tests have become established and are widely used in medical fields associated with the disorder, these tests are often based on unreliable evidence. Moreover, these tests are typically supervised and evaluated by various people and therefore depend strongly on the particularities of the subjects. The experience, level of training, and preconceptions of medical advisors can play an important role in the outcome of the evaluation. Thus, the age of the patients, their physical and psychological states, the duration of the disease, and the specific evolution of the disease are factors that directly affect the reliability of the assessment. In addition, most of these assessments are based on a score combining many elements that is used to quantify the developmental stages of the disease. A drawback of these tests is that a low-resolution scheme is used to rate the results, *i.e.*, the evaluator can only choose among 4 or 5 scoring levels.

Therefore, the diagnosis of PD remains a challenging task. For this reason, we present advances in automatic decision-making systems that can reduce diagnostic error. The most prevalent disorder in misdiagnoses of PD is ET. Nevertheless, other diseases can also be misdiagnosed, such as pseudo-Parkinsonism, vascular disease, and Alzheimer's disease. The primary objective of this study is to improve the differential diagnosis between PD and ET.

Following this brief introduction, we summarize the primary aspects of a clinical diagnosis of tremor. The most common alternatives that have been used to date are presented in Section 2. In Section 3, the DIMETER system is described in terms of the hardware and software used, the proposed tests, and a detailed description of the patterns involved in these tests.

## 2. Clinical Diagnosis of Tremor

Over the past several decades enhancements in the ability of computers to store and manage large amounts of information have enabled computing techniques to be gradually integrated into medicine, where these technologies can enable medical staff to initiate a particular sequence of actions, set strategies, and determine the consequences of decisions from moment to moment.

Unfortunately, these decisions can have unintended consequences because of the medical practitioner's inexperience with similar situations, leading to incorrect diagnoses and inappropriate treatments. Thus, decisions should be made by considering optimization criteria, which may occasionally involve taking serious risks that have a very low probability of occurrence.

It is estimated that in the United States between 44,000 and 98,000 deaths were caused by preventable medical errors. Although most of these errors were related to therapy, including medication and surgical errors, a significant number of these errors resulted from misdiagnoses [4].

Computers are also used in drug delivery. Thus, decision analysis can be based on modeling and simulations, thereby facilitating the management of a patient's individual data. These techniques can be used for dosage adjustment and to determine the best time to administer a drug based on the patient's individual needs [5].

Computing technologies are useful tools for medical decision making. There are well-known systems [6–11] that have harnessed these types of technologies for the diagnosis, characterization, and assessment of PD.

Actigraphs and teleactigraphs (TAGs) are instruments that monitor the activity of the human body by measuring the patient's gross motor activity [12]. An actigraph is typically placed on the wrist, similarly to a wristwatch. The unit continuously records the patient's wrist motion, and the resulting data can be downloaded into a computer for analysis. The teleactigraph is generally used for larger movements and is worn on the shoulder of the dominant arm. This instrument contains a 3D sensor rather than a 1D sensor and has a high sampling frequency and typically a high memory capacity. These instruments can only be used for a few hours (*i.e.*, for short-term use) and are used to determine problems related to walking and other physical disabilities. Other low-cost systems [13] use spectral analyses to measure tremor. Data are captured by an accelerometer, amplifier, and microcomputer with a data acquisition system. A fast Fourier transform (FFT) is used to transform the data. This technology can be used to identify several types of clearly identifiable and quantifiable tremors, *i.e.*, physiological, essential, and other pathological tremor types related to the nervous system. The systems based on these technologies continue to evolve [14,15], and new techniques are being used to detect, classify, or quantify tremor [16,17].

Electromechanical devices can detect clinical manifestations of motor activity disorders by using a mechanical device to perform specified tasks [18].

Other noninvasive technologies that can be used to record the behavior of the subject are based on the electroencephalogram (EEG), magnetoencephalogram (MEG), or electromyogram (EMG) [19]. The microelectrode recordings (MER) technique is used to record the activity of individual neurons, and requires a microelectrode for microstimulation to enhance its performance because the most appropriate targets can be detected for deep brain stimulation (DBS). One of the most surprising recent discoveries indicates that when two signals are obtained from the same patient by different methods (e.g., MER and EMG), the signals can be quite similar in terms of one parameter (e.g., the frequency) but that no two signals are entirely consistent because of variations in the shape of the signal over time.

This result suggests that tremor is caused in several places or decoupled from unknown sources. Gusev [20] developed a phase measure that was more precisely coupled. In [21], a method is presented for frequency tracking in which the extended Kalman filter (EKF) is used to estimate the instantaneous tremor frequency pulse trains detected by MER [21].

Other type of diagnostic systems has been developed for medical imaging. In [22], an automatic diagnostic assistance system is presented to differentiate Alzheimer's disease from mild dementia with Lewy bodies using conventional axial positron emission tomography (PET). Various medical imaging techniques have been developed that can obtain extremely valuable information about diseases related to the nervous system. Being able to “see” regions of the brain in advance of the patient's death (*i.e.*, without performing an autopsy) is an undisputed breakthrough in this field. Several studies of medical imaging reveal a pronounced loss of striatal dopamine carriers in patients with PD [23,24]. Experiments [25] have demonstrated that transcranial ultrasound imaging and single photon emission computed tomography (SPECT) are useful tools in diagnosing PD. Other studies [26] have demonstrated when these techniques (SPECT) are more appropriate for detecting pathological levels of metal accumulation over other neuroimaging techniques, such as computed tomography (CT) or *magnetic resonance imaging* (MRI). In patients with PD, the *substantia nigra* can be described as a well-defined echogenic area, thereby enabling an early diagnosis of these movement disorders. In [27], a SPECT-based method is presented for aiding decision making in the diagnosis of PD.

Extensive studies on measuring general voice disorders have been conducted to diagnose PD. These studies consisted of standard speech tests in which recordings are made using a microphone. The recorded voice signals are analyzed using algorithms to detect certain properties. Recently, various measurement methods have been developed to assess the symptoms of dysphonia. Various methods for PD diagnosis using voice signals are compared in [28].

Advances have also been made to extract data from patients with PD using computer vision (CV) technologies and techniques. A measurement technique for analyzing a patient's motions with a precision movement analyzer using markers and a CDD sensor camera is presented in [29]. In [30], the measurement system uses laser lines and a CMOS image sensor. The system detects the vibration of the back of the hand of a subject in two situations: when the hands are at rest and held in a specified position. In this system, the back of a hand is marked using a diode laser, enabling the detection of the shape, frequency, and relative frequency of the vibration waves of tremor.

## 3. DIMETER System

### 3.1. System Description

DIMETER is a system that is used to objectively characterize tremor patterns by the application of virtual forces. This invention responds to a system of devices and procedures that objectively assess tremor in a person's extremities (hands or fingers). The system records the 3D movements of a person's limb (*i.e.*, in terms of the position, velocity, and acceleration) while he performs a series of tasks that are specified by the system. The system can apply controlled virtual forces to modify the movement to evaluate the effects of static friction or other forces, thereby providing morphological data and frequency criteria for tremor evaluation.

**Figure 1.**
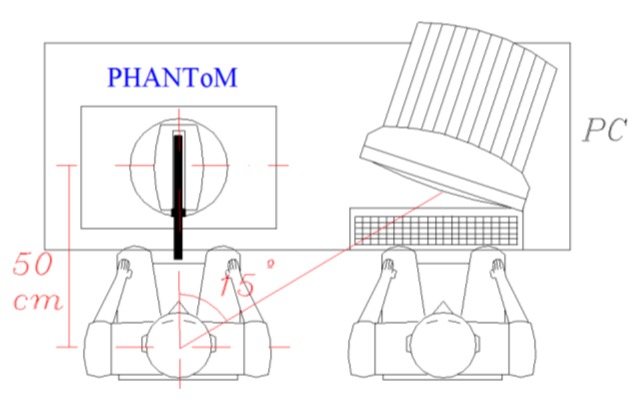
System used for objective characterization of the tremor by applying virtual forces: the patient operates the PHANToM device, and the supervisor operates the computer.

The system consists of a haptic device, computer, and series of procedures that are used to accomplish several tests that are specifically designed to characterize physiological and pathological tremor (Figure 1). These tests are based on the patient's performance during the execution of various motion patterns using his hand or fingers. The system uses motors to apply controlled forces to the patient's upper extremity during the tests. The system also relies on sensors that are placed at the system's joints (in contrast to other systems in which a great number of sensors are used) to record the spatial components of the motion to objectively quantify the specific magnitudes of tremor.

Thus, the system can perform a high-precision 3D motion capture to evaluate tremor in hands or fingers without using inertial sensors (*i.e.*, accelerometers and gyros). The user or patient whose tremor is being assessed moves the articulated mechanical system while holding the end of the aforementioned device.

The electric actuators in the joints of the device are used to measure the effect of virtual forces or loads exerted on the limbs of the subject in different situations, such as in a rest or static position. These forces are applied while performing a spatial movement that is known *a priori*, (*i.e.*, writing or drawing), trying to follow a 3D target or moving randomly or toward specific points.

The tests consist of a series of visual patterns that are displayed on the computer screen. Each pattern is associated with virtual forces or loads that are artificially generated by a control computer. The system continuously records the 3D positions of the hand that correspond to the patient's motion.

The system also consists of specific software, which the supervisor can use to select the test pattern using different windows and menus that are easy to understand. The temporal evolution of the patient's motion can also be tracked using this software. The software can be used to create different files for each session and to store data, such as the patient's personal identification data and the date and time of the test.

Supplementary entries, such as the test conditions, the patient's condition and medication, and the time since the completion of the last test, are also considered to be important information and are therefore stored for each session.

**Figure 2.**
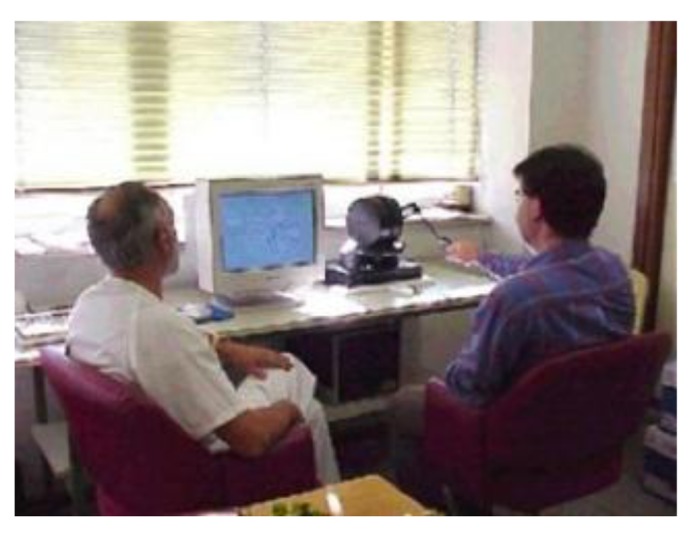
Example of use of the DIMETER system.

**Figure 3.**
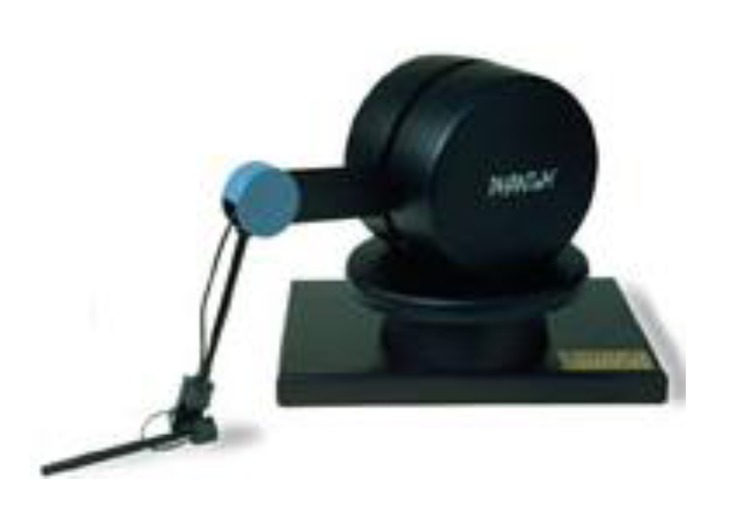
PHANToM device.

DIMETER (Figure 2) uses a device known as PHANToM (Figure 3) manufactured by SensAble Tech (Woburn, MA, USA) that acts as a master. As previously mentioned, this device captures the spatial position and orientation of a solid under the action of different forces.

### 3.2. Test Description

The method presented in this work uses baseline information that was collected using the DIMETER system at two hospitals (Hospital La Princesa and Hospital Ramón y Cajal of Madrid, Spain). This method enables classification tests to be performed on a large number of subjects. The tests were performed on more than 50 patients. In addition, many tests were performed for each subject considered in this study, thereby increasing the number of samples used in the sorting process.

Therefore, the results presented in this work are highly relevant in practice, in contrast to other experimental results that have been reported in the literature in which very good classification results were obtained but the set of subjects was small.

Electromechanical devices, such as the DIMETER system, can also record the movement of a patient's upper limbs accurately and reliably, primarily because this well-known technology has been developed in such areas as robotics. The aforementioned device has an accuracy of 0.03 mm, which provides a reliable record of the patient's movements.

An electromechanical device is more robust and easy to use than other systems that require calibration prior to use (e.g., inertial devices, voice recordings, or computer vision systems that require camera calibration to accurately reconstruct the scene). However, systems dedicated to PD or ET (e.g., [12,13]) are typically focused on tremor evaluation instead of diagnosis.

Thus, this study demonstrates the effectiveness of the DIMETER system for performing a differential diagnosis between PD and ET motion disorders, thereby making a contribution to the diagnosis field. Another significant advantage of DIMETER is related to its ease of use; the use of this system is neither invasive nor uncomfortable for the patient because substances (*i.e.*, markers and radioisotopes) do not need to be administered to the patient to obtain a medical image. Thus, the user only needs to be seated before the system and to use his limbs to move the electromechanical device.

DIMETER is also easy to transport because its size and weight are smaller than those of other technologies, such as medical imaging systems. DIMETER does not require any recalibration after transportation. The only issue with DIMETER that must be considered is that the reference system must be positioned relative to the recording device to properly accommodate the subject for testing. Nevertheless, these drawbacks are less cumbersome than those of systems that use computer vision, for example, which require an extremely precise location and orientation of the cameras involved.

### 3.3. Test Procedure

The test procedure is detailed below.

**Figure 4.**
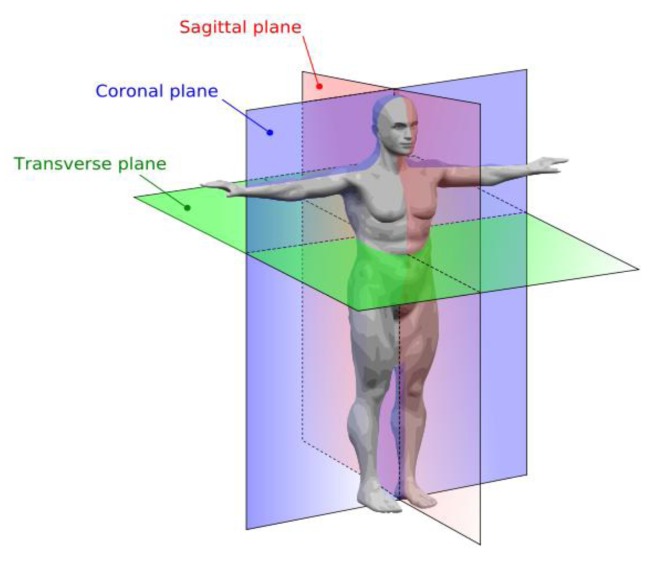
Diagram showing the three major planes of the body.

**Figure 5.**
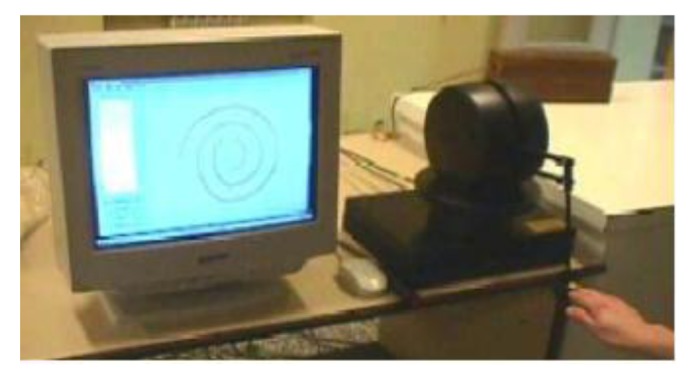
Example of movement test displayed on a computer monitor.

- If the patient is being tested for the first time, a reference ID is created to identify the patient for further analysis and to track the evolution of the patient's test results. If the patient has been previously tested, the existing patient “ID” is selected instead.
- A brief medical examination of the patient is conducted. Comments that the doctor considers to be significant are included in the “Remarks” field to better understand the test results.
- The patient must be correctly positioned relative to the system to be able to completely perform the movement required for the test. Thus, the coronal plane is formed by the X- and Y-axes of the reference system, with the Y-axis pointing upward. The sagittal plane is formed by the Y- and Z-axes, and the transverse plane is formed by the X- and Z-axes (Figure 4).
- The end portion of the measuring system is coupled to the index finger of the patient. The patient's finger should be raised to his shoulder level to leave the field of action of the PHANToM free. The patient should maintain his hand orientation with respect to the reference system used during the test run.
- Verbal instructions are given to the patient on how to perform the proposed test pattern. The objective for using each pattern, which is displayed on the screen and performed using a 3D structure, should be explained to the patient.
- The patient is asked to perform several test exercises for the same pattern. Thus, the patient becomes familiar with the measurement system and all of the proposed exercises. In this manner, the patient becomes aware of the difficulty of the tests.
- Finally, the patient performs the tests, and valid results are obtained (Figure 5).

All of the tests were carried out under the supervision of a team of medical doctors and engineers who were able to invalidate tests because of changes in the patient's position, an increase in the patient's anxiety, or a large disparity between the obtained results and those obtained previously.

A brief resting period was taken after the conclusion of each test. During this time, the technical staff validated the data and made the necessary selections to prepare the pattern for the next test. If the patient became fatigued, the resting period was extended. The patient performed each test using both hands, always starting with the hand that displayed a greater impairment because of tremor.

The tests were performed in the following order: static patterns, kinetic patterns, 3D patterns, and dynamic patterns. All of the tests were videotaped.

To obtain more robust results, all of the tests were conducted in the morning, beginning at 10:30 am. The average duration of the tests was 30 min. The tests that were carried out using the hand with a greater degree of tremor required a longer duration.

### 3.4. Design of Test Patterns

The objective of the DIMETER tests was to normalize and standardize the tests for tremor diagnosis; thus, the standard tests used in conventional clinical evaluations of patients were the bases for designing the patterns (e.g., following horizontal lines on paper, stretching the arm at the shoulder, flexion and extension movements of the arms, and monitoring sinusoidal lines).

The patient may not be proficient in using a computer because of tremor, which can make a mouse very difficult to use. Moreover, patients typically have poor spatial association between their hand movements and the corresponding representation on the screen.

The group of initial basic tests was extended to utilize the dynamic features of PHANToM. Thus, three types of tests were designed: static, kinematic, and dynamic.

The static tests required the patient to remain in a fixed position during the evaluation to assess postural tremor. The kinematic tests focused on evaluating tremor during movement. Finally, the dynamic tests were used to evaluate tremor when external constraint forces were applied. The two latter tests were used to evaluate intentional tremor.

Various tests were designed without the use of the computer screen to evaluate tremor during 3D movements that required the use of several joints while ensuring that the patient remained comfortable. For example, the patient was required to follow a line that forced him to flex and extend his arm.

In addition to prescribing the paths that the patient should follow, the effect of mechanical restraining patterns, such as constant forces or spring and viscous friction forces, on tremor were also evaluated.

The application of restraining forces as a means of tremor suppression in patients has been extensively studied with positive results. Viscous friction forces are particularly effective for suppressing tremor and the inertial forces arising from tremor. Viscous friction forces are particularly effective at low frequencies. In this study, we investigated the combined effects of inertial forces and elastic and viscous friction forces.

These parameters can be varied to obtain the specific mechanical impedance for each type of tremor, thereby tailoring the characterization and evaluation process to individual patients. Therefore, the filter implemented in the interface device for the computer must be tailored to each patient.

Thus, an assessment based on these types of parameters and variables can be used to classify each patient in terms of his mechanical impedance. The staff can then use this parametric information to plan different daily activities for the patient. Table 2 summarizes the types of tests involved in a full diagnostic program, indicating the type of tremor that is evaluated in each case. The patterns that are used for the analyses are described in the following sections.

#### 3.4.1. 2D Patterns

All of the patterns should be performed with the subject seated in front of the device with his arm fully stretched out in front of him/her at shoulder height. The patient should perform the test for both hands.

**Figure 6.**
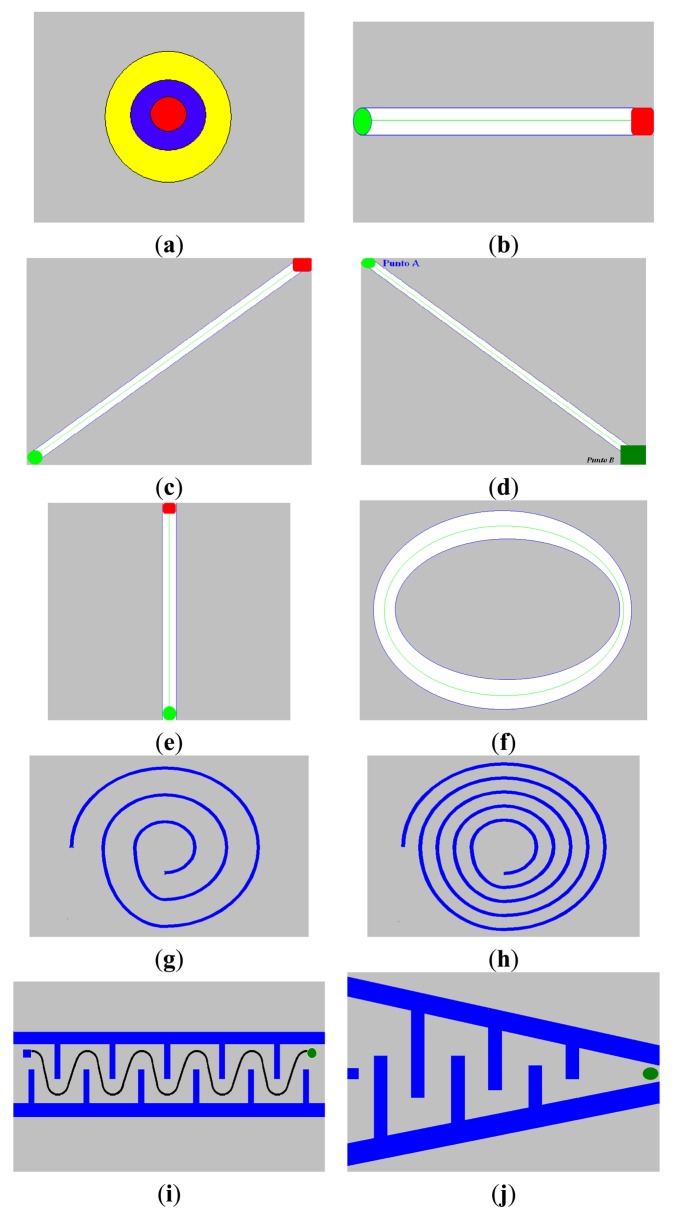
(**a**) pattern 2, (**b**) pattern 3, (**c**) pattern 4, (**d**) pattern 5, (**e**) pattern 6, (**f**) pattern 7, (**g**) pattern 8, (**h**) pattern 9, (**i**) pattern 10, (**j**) pattern 11.

The following tests are performed (Figure 6):

- **Static type tests:**
  ◦ *Pattern 1*: the objective here is to keep the hand relaxed with the elbow on the table and with a 90° between the arm and forearm. The measurements recorded are the three positions, *i.e.*, X, Y, and Z, over time.
  ◦ *Pattern 2*: involves a target, *i.e.*, a point on the screen. The objective is to keep the cursor fixed over a red area on the screen. The measurements recorded are the deviations in X and Y from this point with time.
  Pattern 1 is introduced to analyze the tremor at rest. Pattern 1 is included in the test battery because this type of tremor is very common in patients with PD. Pattern 2 was used to make the components of the postural tremor more evident while maintaining a fixed position with respect to the gravitational force during the test. Usually, this type of tremor implies that patients have severe functional limitations. This type of tremor usually appears when the patient adopts fixed configurations in his limbs to maintain a static position.
- **Kinematic tests:**
  ◦ *Pattern 3*: consists of a horizontal line on the screen. The objective is to move the cursor along a “path” (*i.e.*, a horizontal straight line) between the starting and ending points of the line. The measurements recorded are the deviation from the path (*i.e.*, the distance from the cursor position to the nearest point of the line).
  ◦ *Pattern 4*: the path is a sloping line. The path starts at the bottom point of the line, which forces the patient to perform a down-up transversal movement. The stored data are the same as for the previous test.
  ◦ *Pattern 5*: is similar to pattern 4 except that the respective movement is up-down; therefore, the starting point is the highest point of the path.
  ◦ *Pattern 6*: is a down-up vertical line.
  ◦ *Pattern 7*: is an elliptical line on the screen. The objective is to move the cursor along the circular path. The measurement recorded is the deviation from the ideal path of motion (*i.e.*, the central circumference).
  ◦ *Pattern 8*: is a spiral on the screen. The objective is to follow the spiral beginning from the outside (following the central line as closely as possible). The recorded variables are the X and Y positions on the screen.
  ◦ *Pattern 9*: is a similar, but narrower, spiral path than Pattern 8. The objective is to follow the spiral. The recorded variables are the X and Y positions on the screen.
  ◦ *Pattern 10:* is a sinusoidal curve. The objective is to follow the line from its starting point to its ending point. The X and Y positions on the screen are recorded.
  ◦ *Pattern 11:* is a graph with variable amplitude. The objective is to trace a path from the outside to the inside of the graph. The X and Y positions are recorded. The patient can be asked to perform more precise movements simply by varying the amplitude of the angle between the two borders.

The paths and shapes of the kinetic patterns are used to simulate the effect of intentional tremor in the tests. Different paths have been created to allow for further analysis, e.g., if there is a significant difference between the paths depending on the primary direction of the motion or if there are differences among simultaneous movements in a plane (2D motion). Thus, patterns 3–6 allow a primary direction to be fixed for a task, which allows the impact of a simple objective to be studied for the tremor. However, patterns 7–11 allow an action that combines two primary directions. These tasks are more difficult than the other tasks but introduce the effect of combined movements into the study. Moreover, pattern 9 tests dexterity to evaluate patients whose movements are less restricted by tremor.

Intentional tremor becomes important and can be fairly restrictive in ET patients when they try to achieve a certain position (*i.e.*, pick up a small object) with high precision.

#### 3.4.2. 3D Patterns

**Figure 7.**
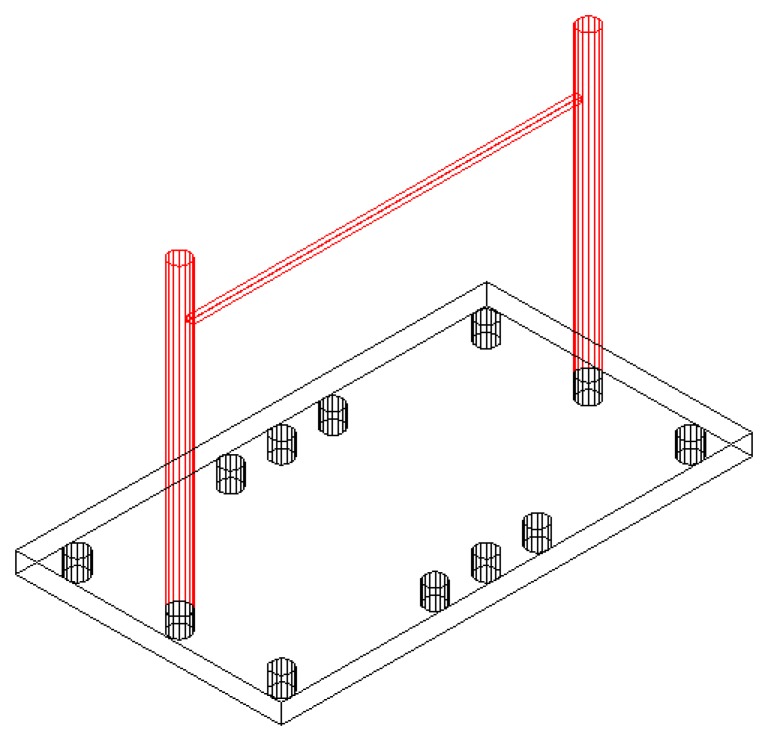
Platform for 3D patterns.

The 3D patterns are a series of straight lines in real space: following these lines requires movement along three axes using the base platform shown in Figure 7. The 3D patterns used in the test are straight lines and are defined below:

◦ *Pattern 12*: is a line parallel to the coronal plane of the subject. The objective is to follow the line from its start to end point. The recorded measurement is the spatial deviation from the ideal path. This line is 40 cm long.
◦ *Pattern 13*: is similar to pattern 12 but is parallel to the sagittal plane of the patient. This line is 20 cm long. The patient must extend and flex his arm at shoulder level during this test.
◦ *Pattern 14*: the subject must perform a movement in all planes in space. The objective and the recorded variables are the same as for the previous patterns. This line is 48 cm long.

The 3D patterns are used to include a depth component in the kinetic study while trying to simulate daily activities more accurately. The primary dysfunctions and disabilities of patients become evident during these activities. The use of tangible 3D objects enables doctors to observe tremor characteristics in response to real stimuli that the patient can touch (*i.e.*, beyond the computer screen). This approach introduces more difficulties into the tests but is more realistic than a computer-based scheme.

#### 3.4.3. Patterns of Virtual Forces

Tests can be conducted using the previously described patterns where mechanical constraints are applied using forces to facilitate patient classification. The forces act as disturbances during the test. The following types of forces are applied.

- A *constant force* is applied consistently during the test. A force up to 5 N (which corresponds to a 500 g force) can be applied in any of the three spatial directions.
- A *spring force* is applied according to the classical spring model that is governed by the following equation:
  (1) F=K⋅X
  where *F* is the force in N, *K* is the spring constant expressed in N/m, and *X* is the displacement with respect to the equilibrium point of the spring (e.g., the distance to the target or path) in m. These types of forces are used to quantify the effort exerted by the patient during the test. Thus, the patient must apply a stronger force to correct the deviation as the deviation from the ideal trajectory becomes greater.

**Figure 8.**
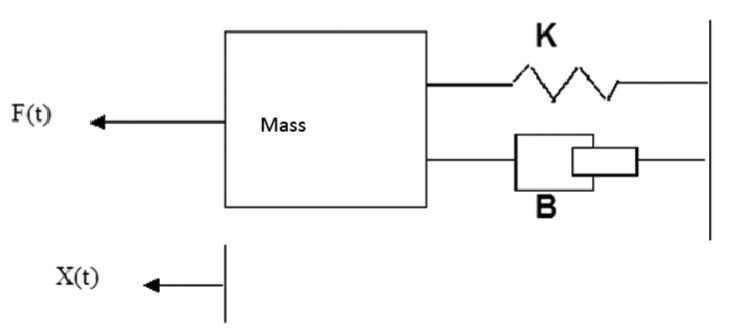
Model of forces exerted on the test mass.

These forces can be progressively applied during the tests to increase the elastic spring constant values within defined limits to obtain a value for *K* that minimizes the tremor amplitude. The value of *K* in the tests ranged between 0 and 0.0005 kg/s^2^. The inertial forces and viscous friction forces can be simulated using the model illustrated in Figure 8:which is governed by the equation given below:

(2) $$F(t)=M\frac{d^{2}x(t)}{dx^{2}}+B\frac{dx(t)}{dx}+Kx(t)$$

where *B* denotes the viscous friction coefficient (kg/s) and *K* (N/m) denotes the spring constant. *X*(*t*) is the displacement of the mass. This equation has three components. The first component represents the inertia of the mass; the second component is the friction force, which depends on the speed; and the third component is the elastic force, which is proportional to the displacement.

These factors combine to produce a viscous sensation and a slight sense of inertia that prevent the range of motion from being increased, whereas the motion is not restricted in any specific direction but adjusts to the movements. The value of the applied force in the tests corresponded to the inertia of a mass of 0.05 kg and a viscous friction coefficient of 0.0015 kg/s.

The following dynamic patterns were tested, including the forces:

◦ *Pattern 15*: which is equivalent to pattern 2 but was used with a constant force of 5 N in the Y-direction (*i.e.*, downwards)
◦ *Pattern 16*: which is equivalent to pattern 3 but was used with increasing values of *K* in the Y-direction (*i.e.*, perpendicular to the movement)
◦ *Pattern 17*: which is equivalent to pattern 3 but includes inertia and viscous friction
◦ *Pattern 18*: which is equivalent to pattern 8 but includes inertia and viscous friction
◦ *Pattern 19*: which is equivalent to pattern 13 but includes inertia and viscous friction factors

The dynamic patterns highlight and justify the use of a haptic element as the kernel of the system. Thus, the system can generate extreme forces to detect differences in the patient's behavior (which depend on the tremor disorder) when external actuations are applied [31].

The disparities between a patient's execution of a kinematic pattern and its corresponding dynamic pattern reveal symptoms that are relevant to the type of tremor because postural tremor or rest tremor can be attenuated by the presence of gravitational or viscous inertia in many cases.

The tests that were conducted to define and select the patterns used in this work were performed in close conjunction with the medical staff of Ramón y Cajal Hospital, a world-renowned center for the treatment of PD, and the Hospital La Princesa, which are both located in Madrid. The help and support of the neurological and neurophysiological teams of the Hospital Ramón y Cajal were critical during the tests conducted in this facility. The neurological department of La Princesa Hospital played a fundamental role in defining the test protocols.

The patterns were derived from actual protocols used in diagnosis: the variety of these protocols was increased, and the protocols were modified to utilize the capabilities of the haptic device, primarily performing 3D motion capture and receiving force feedback.

## 4. Characterization of Human Tremor by DIMETER

As previously mentioned, the primary goal of this study was to characterize each class of tremor: Parkinsonian tremor, ET, and physiological tremor. Tremor recognition is not as simple as many other automatic classification schemes that operate in highly structured environments. The primary difficulty is that tremor classification involves human beings (in this case, patients)

The environment for manufacturing tasks and other similar actions is typically extremely rigid, primarily because industrial processes require this manner of operation. However, a non-static environment causes the difficulty of performing classification to increase enormously. This situation is encountered in tremor classification, where recordings of clinical manifestations of motion disorders may result from several diseases and may depend strongly on the state of the patient (*i.e.*, age, state of mind, treatment received, other diseases, time of day, eating habits, and the stage and evolution of the disease) and the evaluator (*i.e.*, the type and level of education and experience).

Moreover, tremor can be observed in both healthy subjects and PD patients, and the state of the tremor changes throughout the day (*i.e.*, because of freezing, rigidity, and bradykinesia). These factors explain the large numbers of misdiagnoses in PD, even when qualified expert personnel are involved in the diagnosis process.

In this study, a large and complex set of parameters was used to obtain as much information as possible. These parameters contained statistical information on the motion test performed by the subject, such as the power spectral density (PSD) of the tremor and statistics associated with the trajectory.

The device used in DIMETER enables a high-frequency positional sampling (100 samples per second), thereby measuring the patient's movement with time along each spatial Cartesian axis with high accuracy.

The PSD measures the power distribution generated within the band of frequencies. This information is particularly relevant for classification. Thus, the calculated PSD [2] can be used to detect the bands that contain the most power. Important information can be obtained from the movement of the subject in parametric form.

The choice of these parameters was inspired by time-series concepts. Physiological tremor in patients can often be modeled as a linear stochastic process [32,33], whereas tremor in Parkinsonian patients can be modeled as signals of a nonlinear chaotic nature [34]. The features of ET lie in between the two aforementioned tremors.

Using previously stated assumptions, high-order statistics (HOS) were combined with conventional statistics and the PSD values (*i.e.*, parameters from 1 to 9) to extract up to 26 parameters for each series (Table 3). This method allowed vectors with 26 dimensions to be processed. The following table describes these parameters and the function or method used to obtain them.

The trembling motion in PD is a signal that is characterized by non-stationary or transient features, such as tendencies, abrupt changes, and start and stop events. There many trajectories in which PD subjects exhibit rigidity or great changes during the performance of the test. In most cases, it is useful to apply a Fourier transform to the signal. Thus, in 1946, Denis Gabor modified the Fourier transform to analyze a small section of a short time interval (*i.e.*, corresponding to a time window). This modification is known as a short-time Fourier transform (STFT) and transforms the signal into a bi-dimensional space in the time interval and frequency. However, this information can only be obtained with limited accuracy because of the length of the window that sets the resolution.

## 5. Tremor Classification Using MLP

In this study, we developed a classifier based on a multilayer perceptron (MLP) neural network to classify motion disorders. Therefore, the size of the parameter vector (in this case, 26) determined the dimension of the input. However, the dimension of the output layer was equal to the number of classes (*i.e.*, class 1 was identified by the vector (1;0;0), corresponding to an ET patient; class number 2 was identified by the vector (0;1;0), corresponding to a PD patient; and class number 3 was identified by (0;0;1), corresponding to a healthy patient).

The number of hidden neurons for the hidden layer was determined experimentally. The activation function selected for both the hidden and output layers was a hyperbolic tangent sigmoid (tansig).

A total of 364 vectors were used to train the network, and 156 vectors were used to validate the network. The cases used in the test had the following distribution: 210 healthy patients, 34 ET patients, and 120 PD patients (as given by the medical records). Ten training simulations were conducted for each group of tests, as described below.

A back-propagation algorithm was used in the training. The input vectors were previously normalized by applying the method of zero mean and a standard deviation of 1. Two hundred training epochs were selected, which was considered to be optimal because a larger number of epochs could have caused overtraining but a smaller number could not provide the necessary information for the interpolation.

Several tests were designed in which a variable number of parameters were used following the classification given below.

**Figure 9.**
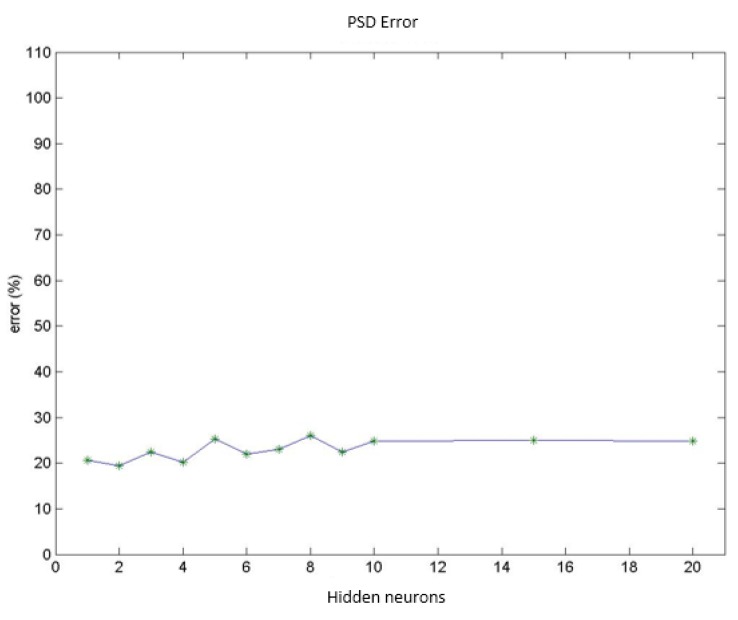
Diagnosis error (mean value) obtained using only PSD parameters.

**Figure 10.**
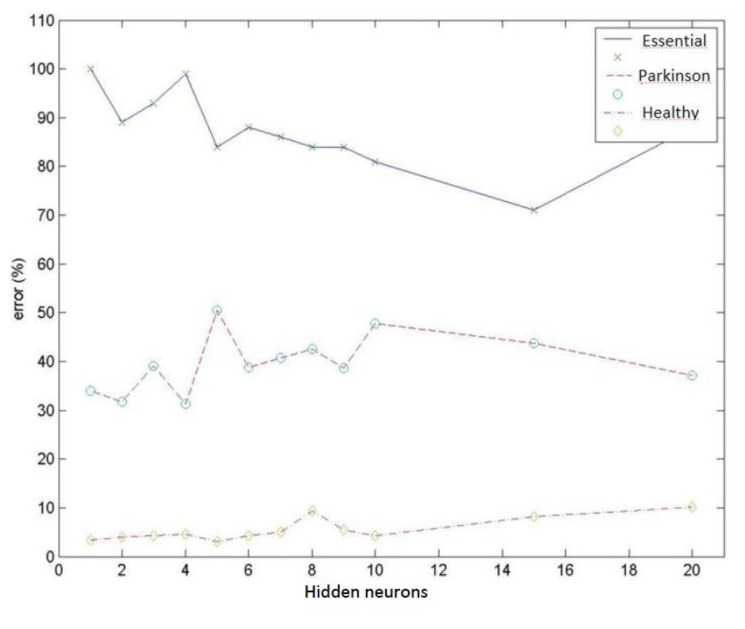
Error distribution for each type of pathology obtained using PSD parameters.

**Figure 11.**
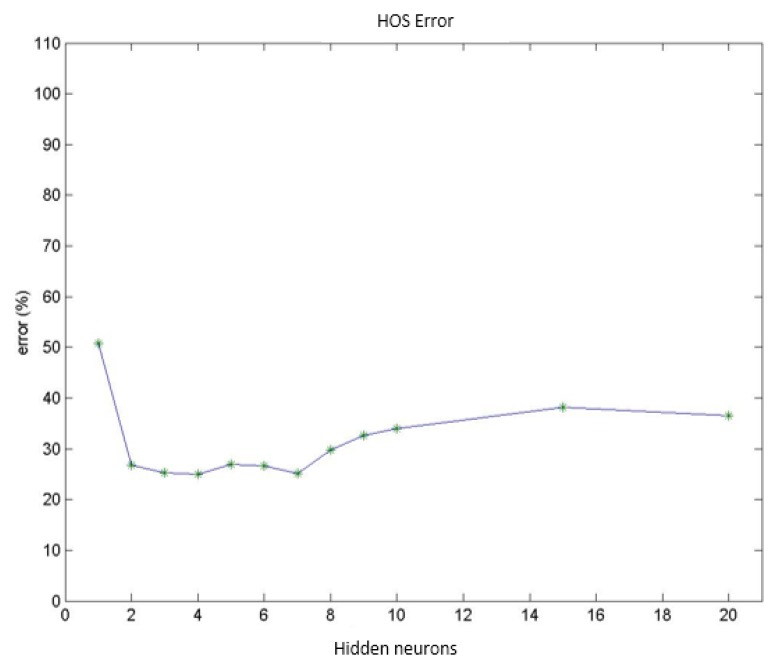
Diagnosis error (mean value) obtained using HOS parameters.

**Figure 12.**
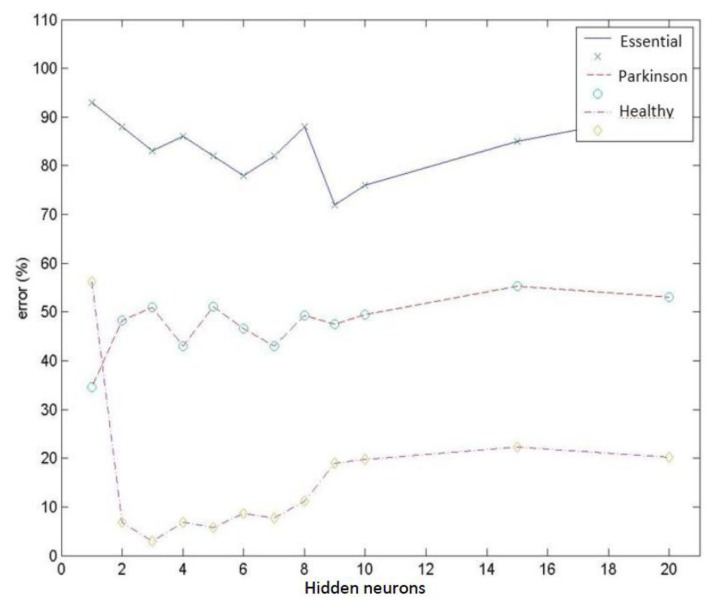
Error distribution for each type of pathology obtained using HOS parameters.

**Figure 13.**
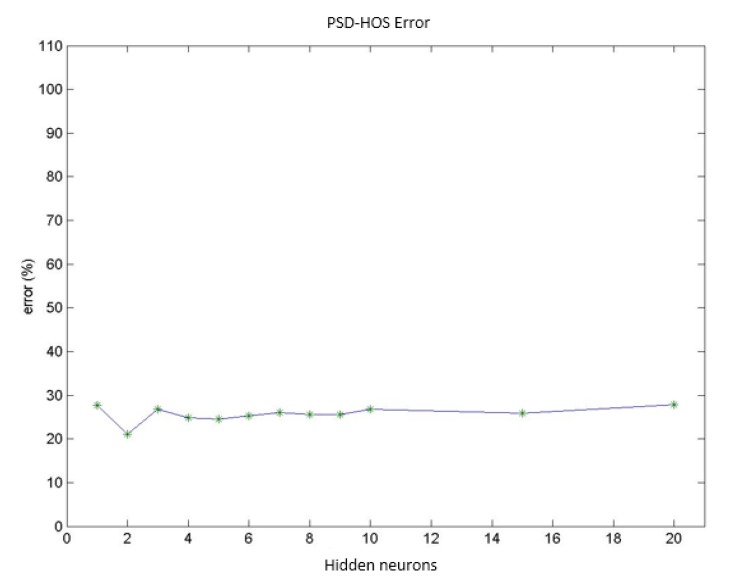
Diagnosis error (mean value) obtained using all parameters.

**Figure 14.**
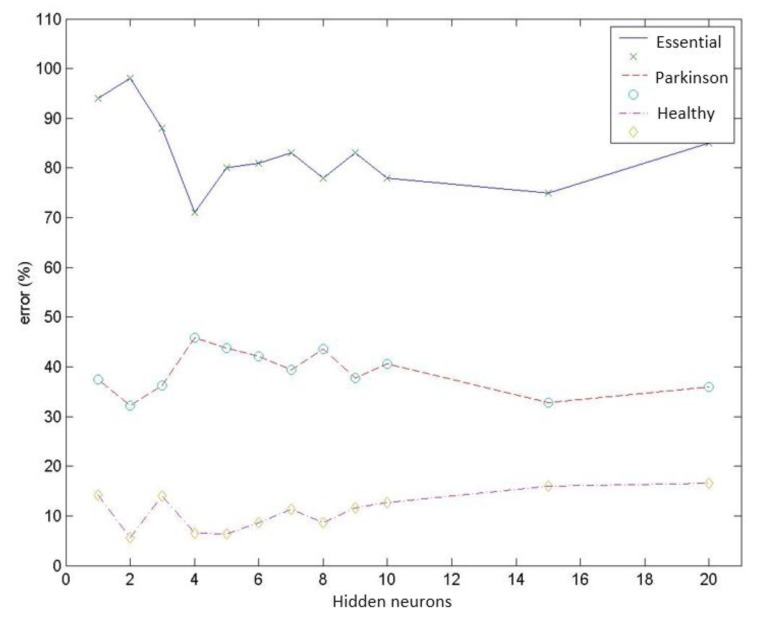
Error distribution for each type of pathology obtained using all available parameters.

- Test group 1: Only the PSD parameters (*i.e.*, parameters 1 to 9) were considered in this group. In this case, the network reached a minimum classification error of 19.4% using a group of 156 vectors (which corresponded to different patient cases) for networks with 2 neurons in the hidden layer. Increasing the number of neurons stepwise increased the error by up to 24.0% for 10 hidden neurons. Figure 9 presents the mean value for the misdiagnoses as a function of the number of hidden neurons. Figure 10 presents the error distribution for each type of pathology using this group of parameters.
- Test group 2: Only the HOS parameters (*i.e.*, parameters 10 to 26) were used under the same conditions as for group 1. The minimum error was close to 24% (using 4 hidden neurons for the MLPs), which was higher than for the previous case. Considering only the error decomposition, most of these errors (86%) occurred for ET patients, primarily because very few ET cases were available for training, and only 8% of the error occurred for healthy diagnoses. Figures 11 and 12 present the mean error that was obtained by varying the number of hidden layers and the error for each type of pathology.
- Test group 3: All of the parameters (*i.e.*, the 26 parameters) were used in this group. In this case, an error of approximately 24% was consistently obtained starting from 4 hidden neurons. In this case, 70% of the error cases occurred for ET patients. Figures 13 and 14 illustrate the results that were obtained in the same manner as for the previous cases.

In the first analysis, the mean values obtained from each test are compared to each other. Thus, similar mean values for the errors in diagnosis were obtained in all of the cases, especially for cases 2 and 3, (e.g., 24% using 4 hidden layers). This conclusion indicated that the HOS and PSD parameters could be used to obtain the same results independently of each other, *i.e.*, fewer parameters could be used to perform the diagnosis. Nevertheless, better results were obtained when a more detailed analysis was performed and the errors were categorized according to the respective pathology, particularly for ET (for which fewer cases were available for training). The error contribution from ET reached 70%, which was the lowest error obtained for all of the tests using this configuration.

These results motivate our next objective, to find the minimum number of required characteristics and hidden neurons that will produce an acceptable classification error. Decreasing the number of vector components and neurons in the hidden layer would accelerate the classification and produce a simpler simulation model that could be used to reduce the classification error in future studies using other methods.

Neurophysiologists, neurologists, and neurosurgeons are clearly interested in using a system that can improve the diagnosis of the diseases considered in this study while being scientifically based, *i.e.*, a method that does not rely solely on personal perception and that is based on objectively quantified data.

## 6. Conclusions

Current tests and diagnosis are based on qualitative scales that are highly dependent on the subjective perception of the individuals that oversee the diagnosis. Therefore, the main advantage of the diagnostic aid system developed in this study is its ability to produce repetitive and objective indicators of a patient's state from several tests based on patterns that are specifically designed to evaluate each aspect of the tremor.

As previously mentioned, a distinguishing feature of the DIMETER system compared to other tremor detection systems is its capability of introducing active forces into the tests. This characteristic improves diagnosis by enabling important behavioral features to be distinguished.

Moreover, the system enables motions to be recorded for further analysis to compare results independently of external or subjective factors that can be highly variable.

The system facilitates the diagnosis and enables the patient's history to be more effectively monitored, thereby storing all of the available tests for evaluation upon demand.

The results indicated that the evolution of a patient's disease resulted in variable behavior for a given pathology. Therefore, the primary characteristics of a patient's motion can conceal or even overlap with other pathologies.

Methods based on PSD, HOS, and neural networks contributed an average classification error of only 20% (considering the proportion of vectors of each class) at the stage that validation information was not introduced into the training process. This error is admissible given the limited of number of feature vectors of ET. HOS enabled greater discrimination between ET and PD but did not decrease the error in differentiating between physiological tremor and pathological tremor.

According to the *Canadian Journal of Neuroscience*, general neurologists treating tremor have a diagnostic error rate of 25%–35% [35]; therefore, the results obtained using the system developed in this study can be considered good, and the system in its current state would be a useful diagnostic tool.

Unfortunately, the analysis of results indicated that the characterization brackets for the case studies were not far away from each other and often strongly overlapped, thus hindering classification.

These results indicate that the information used to categorize a group of interest is not intrinsically contained in the individual characteristics but in the coupling between these characteristics. Therefore, the analysis of the covariance among these characteristics would yield better results. Thus, the authors envision multi-variable classifiers as the best way to address the problem. This conclusion motivates future research directions.

Doctors with whom these results were discussed also reported that some patients were misdiagnosed because of short or imprecise clinical histories. These cases could introduce considerable error into the data and should be identified and removed from the training sets. This consideration of misdiagnosis has resulted in a new research direction that is based on the exogenous variability in patients and their clinical history. These datasets should be considered outliers that make the training less precise. Therefore, these datasets should be automatically detected and discarded from training sets.

Finally, another factor that makes the classification difficult is the difference between the incidence and prevalence of the diseases. These findings demonstrate that it is very difficult to use the same number of patients suffering from each pathology to train the classifiers (*i.e.*, significantly fewer patients suffer from ET than from PD).

In summary, DIMETER is an autonomous system from a facultative perspective. The system fully integrates the clinical tests and the results of the analyses to produce a diagnosis. DIMETER provides doctors with slightly better results than current procedures. However, the objective of the research is to improve data categorization by adding new multi-variable and outlier removal techniques.

## 7. Future Work

As previously mentioned, the detection of outliers is a prerequisite for improving classification and rapidly decreasing diagnostic failures. The use of multi-variable techniques is also expected to provide promising results.

However, the results obtained using frequency component analysis indicate that wavelet analysis is the next logical step following the use of STFT. Wavelet analysis enables the use of variable-size windows: large time intervals for low-frequency data and small intervals for high-frequency data. However, working with vectors of 26 parameters may become challenging if conventional computers are used.

Furthermore, pre-processing based on parameter selection may be difficult to execute because of the large number of possible combinations involved. In addition, the training process of a neural classifier may be computationally expensive when the dimensionality is high (*i.e.*, many parameters are considered). This result is obtained because the training vectors must be introduced into the classifier many times to achieve a good learning process. This result strongly depends on the neural classifier and learning rules that are chosen for the training.

In future work, principal component analysis (PCA) will also be considered with the following objective: we will analyze “t” tests of “n” variables to determine whether the available information can be adequately represented by constructing a smaller number of variables from a linear combination of the original variables.

Another course of action would be to use typical techniques in pattern recognition together with discrete hidden Markov models (HMMs): here, a dissimilarity space based on the distances between samples (sequences) and HMMs is used to obtain feature vectors [36,37] that can be introduced into a neural network for classification. The use of this methodology will also be considered in the context of traditional feature-based classifiers, including linear and nonlinear support vector machines [38].

## Figures and Tables

**Table 1.** Range of prevalence and incidence rates from different European studies [1–3].

	**PD**	**ET**
Prevalence	1.8% to 2.6%	4.05% to 5.00%
Incidence (hab./year)	5/100,000 to 346/100,000	18/100,000

**Table 2.** Pattern classification.

**Type**	**Type of Motion**	**Non-Dynamic**	**Dynamic**
**Static**	No motion	Postural tremor and rest	Postural tremor with external disturbances
	Single joint	Intentional tremor in simple movements	Intentional tremor in simple movements with external disturbances
**Kinetic**	Multiple joint	Intentional tremor in complex movements	Intentional tremor in complex movements with external disturbances

**Table 3.** List of 26 parameters obtained from each test.

**Parameter**	**Description**	**Formula**
1	Maximum PSD value	*max* (PSD)
2	Frequency corresponding to maximum PSD value	*f_max_* (PSD)
3	First moment of PSD	*m*_1_ (PSD)
4	Second moment of PSD	*m*_2_ (PSD)
5	Fourth moment of PSD	*m*_4_ (PSD)
6	Number of spectrum samples with PSD values above 0.72%	*N*_0.72%_ (PSD)
7	Number of spectrum samples with PSD values above 2.42%	*N*_2.42%_ (PSD)
8	Number of spectrum samples with PSD values above 95.3%	*N*_95.3%_ (PSD)
9	Fifth moment of PSD	*m*_5_ (PSD)
10	Sum of bispectrum diagonal values	*S* (diag|bisp|)
11	Sum of bispectrum values	*S* (|bisp|)
12	Sum of logarithms of bispectrum diagonal values	*S* (log(diag|bisp|))
13	Sum of logarithms of bispectrum values	*S* (log(|bisp|))
14	First moment of bispectrum diagonal	*m*_1_ (diag|bisp|)
15	Second moment of bispectrum diagonal	*m*_2_ (diag|bisp|)
16	First moment of logarithm of bispectrum diagonal	*m*_1_ (ldg(diag|bisp|))
17	Maximum value of trispectrum diagonal	*max* (diag|trisp|)
18	Normalized sum of trispectrum diagonal values	*S_r_* (diag|trisp|)
19	First moment of trispectrum diagonal	*m*_1_ (diag|trisp|)
20	Second moment of trispectrum diagonal	*m*_2_ (diag|trisp|)
21	Third moment of trispectrum diagonal	*m*_3_ (diag|trisp|)
22	Number of samples of bispectrum diagonal with values above 0.29%	*N*_0.29%_ (diag|bisp|)
23	Number of samples of bispectrum diagonal with values was above 4.3%	*N*_0.29%_ (diag|bisp|)
24	Number of samples of diagonal trispectrum with values above 0.15%	*N*_0.15%_ (diag|trisp|)
25	Number of samples of diagonal trispectrum with values above 5.6 × 10^−6^%.	(diag|trisp|)
26	Fifth moment of bispectrum diagonal	*m*_5_ (diag|trisp|)
